# Alterations in Blood and Hippocampal mRNA and miRNA Expression, Along with Fat Deposition in Female B6C3F1 Mice Continuously Exposed to Prenatal Low-Dose-Rate Radiation and Their Comparison with Male Mice

**DOI:** 10.3390/cells14030173

**Published:** 2025-01-23

**Authors:** Hong Wang, Ignacia Braga Tanaka, Salihah Lau, Satoshi Tanaka, Amanda Tan, Feng Ru Tang

**Affiliations:** 1 Radiation Physiology Lab, Singapore Nuclear Research and Safety Initiative, National University of Singapore, Singapore 118415, Singapore; 2 Department of Radiobiology, Institute for Environmental Sciences, 2-121 Hacchazawa, Takahoko, Rokkasho, Aomori 039-3213, Japan

**Keywords:** prenatal, low dose rate, chronic radiation exposure, health effect, female mice

## Abstract

Our recent study revealed that continuous prenatal low-dose-rate irradiation did not induce cellular changes in the dentate gyrus of the hippocampus in male B6C3F1 mice exposed to gamma rays during prenatal development. However, changes in body weight, body mass index (BMI), locomotor ability, and mRNA and miRNA expressions in the hippocampus and blood were observed. To investigate potential sex differences in the effects of prenatal gamma irradiation, we conducted a parallel study on female B6C3F1 mice. The results showed significant reductions in the weight of the lungs and left kidney in prenatally irradiated female offspring, accompanied by significantly increased fat deposits in the mesentery, retroperitoneal, and left perigonadal areas. Despite these systemic changes, no cellular alterations were observed in the subgranular zone (immature neurons) or the hilus of the dentate gyrus (mature neurons and glial cells, including astrocytes, microglia, and oligodendrocyte progenitor cells). However, significant increases in hippocampal mRNA expression were detected for genes such as *H2bc24*, *Fos*, *Cd74*, *Tent5a*, *Traip*, and *Sap25*. Conversely, downregulation of mRNAs *Inpp5j* and *Gdf3* was observed in whole blood. A Venn diagram highlighted the differential expression of two mRNAs, *Ttn* and *Slc43a3*, between the hippocampus and whole blood. Comparisons between prenatally irradiated male and female B6C3F1 mice revealed sex-specific differences. In whole blood, 4 mRNAs (*Scd1*, *Cd59b*, *Vmn1r58*, and *Gm42427*) and 1 miRNA (mmu-miR-8112) exhibited differential expression. In the hippocampus, 12 mRNAs and 2 novel miRNAs were differentially expressed between the sexes. qRT-PCR analysis validated the upregulation of *H2bc24*, *Fos*, *Cd74*, and *Tent5a* in the female hippocampus. These gene expression changes may be associated with the increased fat deposition observed following chronic low-dose-rate gamma irradiation exposure. This study underscores the importance of investigating sex-specific biological responses to prenatal gamma irradiation and highlights potential molecular pathways linked to observed physiological changes.

## 1. Introduction

Acute high-dose/dose rate prenatal irradiation may induce pregnancy loss (miscarriage, stillbirth), malformation, disturbances of growth or development, and mutagenic and carcinogenic effects in offspring [1]. However, the effect of acute prenatal irradiation at low doses (<100 mSv) is still controversial [2]. It is also not known whether chronic continuous prenatal irradiation with low dose rates (<6 mGy/h) induces adverse health effects in the offspring. Analysis of cancer incidence and mortality in childhood or adulthood over 60 years (from January 1950 to December 2009) in the Urals Prenatally Exposed Cohort (UPEC), i.e., female workers and residents living in the contaminated Techa River villages showed no strong evidence that chronic prenatal low-dose-rate exposure increased the risk of solid cancers in the offspring [3]. For both incidence and mortality, a tendency towards a decreased relative risk was noted with increasing doses to the soft tissues of the fetus [3]. High-dose-rate prenatal protracted gamma irradiation did not result in the same morphologic alterations, such as derangement of the laminar structure of pyramidal cells within the hippocampus or malformation of cerebellar lobules in the offspring brain as observed after prenatal single irradiation [4]. Protracted gamma irradiation at a high dose rate of 42 mGy/h from embryonic day E13 to E16 (total dose = 3 Gy), however, showed a statistically significant decrease in the numbers of pyramidal and granule cells in the hippocampus and cerebellar Purkinje and granule cells (approximately 50% decrease) in both male and female Wistar rats [4]. Chronic irradiation at a low dose rate of 100 mGy/day (4.55 mGy/h) for the entire gestation period (total dose = 1.8 Gy) did not induce any impairment of neurogenesis in the subgranular zone or loss of hilar neurons in the dentate gyrus of male B6C3F1 mice [5].

Low-dose and low-dose-rate ionizing radiation has become a significant health concern due to factors such as increased use in medical diagnostics, space travel, and nuclear accidents. Notably, substantial sex differences in radiation response have also been documented [6,7,8,9,10,11]. Our previous studies have shown significantly reduced neuropathological and functional changes following continuous postnatal radiation exposure when compared to an acute high-dose-rate irradiation at the same cumulative dose of 5 Gy [12,13,14,15]. The present study aimed to investigate potential sex differences in the effects of prenatal gamma irradiation in B6C3F1 mice. The results of male offspring have been published [5]. A parallel study on female mice was conducted, in terms of cellular, mRNA, and miRNA changes in the hippocampus and blood, and body, organ, and adipose tissue weights in B6C3F1 female offspring continuously exposed to low-dose-rate irradiation in utero. These changes were compared to those found in B6C3F1 male mice similarly exposed in the previous study [5].

## 2. Materials and Methods

### 2.1. Animals and Irradiation Procedure

Six-week-old mice (C57BL/6JJcl females and C3H/HeNJcl males) were purchased from CLEA Japan Inc., Tokyo, Japan, and used as parent stocks as described previously [16]. Pregnant dams continuously received whole-body ^137^Cs gamma irradiation at a dose rate of 100 mGy/day from gestation day (GD) 0 to 18 to a total dose of 1.8 Gy. The absorbed doses by the pregnant dam were measured using thermoluminescence dosimeters (TLDs). Pups were weaned at 21 days, grouped by sex, and housed 5 mice/cage. Age-matched non-irradiated pregnant dams (and their pups) were used as controls. Female mice from the same litters as the male offspring in our previous study [5] were used in the present study. A total of 30 female offspring (n = 15 non-irradiated control and n = 15 irradiated) were housed in SPF environmental conditions at the Low-Dose Radiation Effects Research Facility (LERF) of the Institute for Environmental Sciences (IES), Japan. SPF maintenance and husbandry practices (ad libitum feed and water, 12 h light–dark cycle, weekly cage change, daily health monitoring) were as described previously [17]. The experiments were performed under the Guidelines for Animal Experiments of the Institute for Environmental Sciences in Japan.

### 2.2. Pathological Examination

Mice were sacrificed using carbon monoxide asphyxiation at approximately 1 year of age. Whole blood samples were collected via cardiac puncture with a 1 mL sterile syringe equipped with a 26-gauge needle. The xyphoid process of the mouse’s rib cage was located by tracing downward from the sternum to the center of the ribcage. The needle tip was positioned below the xyphoid process, with the needle bevel facing upward. The needle was then inserted below the sternum at an angle of approximately 30 degrees cranially, directed toward the strongest heartbeat. Once blood was observed within the hub of the needle, the syringe was steadied, and blood was withdrawn slowly and carefully. The whole blood (0.5 mL) was added to 2 mL tubes pre-loaded with RNAlater solution and stored frozen at −80 °C. The mice were then subjected to necropsy (gross examination). Organs except the brain were collected, examined, weighed, and fixed in 10% neutral buffered formalin for histopathological examination based on a standard protocol [5,18]. The whole brain was dissected and separated sagittally into the left and right hemispheres. The right hemisphere was fixed in 4% paraformaldehyde for 24 h, then transferred to 30% sucrose in 0.1 M phosphate buffer (pH 7.4) for immunohistochemistry. The hippocampus was dissected from the left hemisphere and stored frozen at −80 °C until it was processed for RNA extraction.

### 2.3. Immunohistochemical Staining of the Hippocampus

Seven to nine serial sagittal sections (40 μm thick) of the right hemisphere of the brain (n = 5/group) were placed in 24-well-plate with PBS, treated with 3% H_2_O_2_ and Animal-Free Blocker^®^ and Diluent, R.T.U. (Vector Laboratories, Inc., Newark, CA, USA), and then immunostained for newly generated neuronal marker DCX (1: 500; Santa Cruz Biotechnology Inc., Dallas, TX, USA), mature neuronal marker NeuN (1: 1000), oligodendrocyte precursor cell marker PDGFRα (1: 200), astrocyte marker GFAP (1: 200), and microglial marker IBA1 (1: 200) (Cell Signaling Technology, Danvers, MA, USA). The sections were washed and incubated with respective secondary antibodies, avidin–biotin complex (ABC) reagent (Vector Laboratories Inc., Burlingame, CA, USA) and DAB Peroxidase Substrate (Vector Laboratories Inc., Burlingame, CA, USA). Finally, the sections were washed, mounted, counterstained, and covered.

The immunostained sections were viewed and photographed (Leica Microsystems GmbH, Wetzlar, Germany), and a Stereologer System (Stereology Resource Center, Biosciences Inc., Tampa, FL, USA) was used to analyze the number of DCX-immunopositive cells in the subgranular zone, indicated as the number/length (mm); NeuN, PDGFRα, and GFAP immunopositive cells in the hilus; and IBA1 immunopositive cells in the hilus and stratum granulosum, indicated as the number/volume (mm^3^).

### 2.4. RNA Extraction from Female B6C3F1 Mouse Hippocampus and Whole Blood

Total RNAs were extracted from the hippocampus of 6 non-irradiated control and 6 prenatally irradiated mice from the 100 mGy/day group. miRNeasy Mini Kit (Qiagen, Hilden, Germany) was used, and RNA extraction was performed according to the manufacturer’s instructions as previously described [5,15]. Briefly, the hippocampus was homogenized with 140 µL chloroform. After centrifugation, the upper aqueous RNA phase was collected, mixed with 100% ethanol, and added into RNeasy Mini spin column. RNA was eluted from the column membrane by centrifugation and dissolved in RNase-free water.

Mouse RiboPure™-Blood RNA Isolation Kit (Life Technologies Holdings Pte Ltd., Singapore) was used to isolate RNA from whole blood. Frozen whole blood was thawed and centrifuged. The cell pellet was reconstituted by adding the lysis solution and centrifuged. The aqueous upper phase was recovered and vacuum-filtered through a Filter Cartridge. RNAs were eluted with nuclease-free water.

### 2.5. Systematic mRNA Sequencing Analysis and miRNA Sequencing (miRSeq)

mRNA and miRNA sequencing of the hippocampus and the blood from female mice were performed as described previously [5,15].

For mRNA sequencing, RNA samples were denatured, enriched, and fragmented. After the first and second strands of cDNA were synthesized, the double-stranded cDNA was subjected to end-repair. A single ‘A’ nucleotide was added to the 3′ ends of the blunt fragments, followed by adaptor ligation and PCR amplification. Finally, DNA nanoball containing multiple copies of DNA was produced, and sequenced.

For miRNA sequencing, the RNA sample was combined with 3′ and 5′ adapters. The RT-PCR products were purified with PAGE gel. DNA nanoball containing multiple copies of DNA was generated and sequenced through Probe-Anchor Synthesis (cPAS).

DNB SEQ platform (BGI, Beijing, China) was used to analyze the sequencing data. DESeq2 method detected 170 mRNAs and 44 miRNAs which were differentially expressed in the female hippocampus based upon the more-than-1.5-fold change between control and irradiated samples and *p*-value less than 0.05. In total, 41 mRNAs and 3 miRNAs were differentially expressed in female blood samples.

### 2.6. Real-Time Quantitative Reverse Transcription PCR (qRT-PCR) Analysis of mRNA

Maxima first strand cDNA synthesis kits (Thermo Fisher Scientific, Waltham, MA, USA) were used to reverse-transcribe RNA into cDNA as described previously [5,15]. Briefly, 2 µL Maxima Enzyme Mix, 1 µg RNA, 4 µL 5× Reaction Mix, and nuclease-free water composed of 20 µL of the reaction mixture were incubated at 25 °C for 10 min, 50 °C for 45 min, and 85 °C for 5 min.

In total, 20 µL PCR master mix composed of 10 µL 2× Maxima SYBR Green qPCR Master Mix, 2 µL diluted cDNA, 4 µL nuclease-free water, and 2 µL 10× forward and reverse primers for target genes (Table 1 and Table 2). PCR amplification was performed at 95 °C for 10 min, followed by 40 cycles of 95 °C for 15 s, 60 °C for 30 s, and 72 °C for 30 s. Gapdh expression was used as an internal control. A delta–delta CT (∆∆CT) method was used to calculate the fold change in target mRNA expression.

### 2.7. Real-Time qRT-PCR Analysis for miRNA

A miScript II RT kit (Qiagen, Hilden, Germany) was used for RNA reverse transcription [5,15]. In total, 12 µL of template RNA in nuclease-free water, 4 µL of 5× HiSpec buffer, 2 µL of reverse transcripts mix, and 2 µL of 10× nucleotide mix were incubated at 37 °C for 1 h and then 95 °C for 5 min.

A total of 20 µL of the PCR reaction mixture composed of 10 µL of 2× miScript SYBR green PCR master mix, 2 µL of diluted cDNA, 2 µL of 10× miScript universal primer, 2 µL of primer for target miRNAs (Table 3), and 4 µL of nuclease-free water. The mixture was incubated at 95 °C for 15 min and 40 cycles of 94 °C for 15 s, 55 °C for 30 s, and 70 °C for 30 s. PCR amplification and fluorescence data collection were performed on QuantStudio 6 Real-Time PCR Systems (Thermo Fisher Scientific, Waltham, MA, USA). miR-68 was used as an internal control. The delta–delta CT (∆∆CT) method was used to calculate the fold change in target miRNA expression.

### 2.8. Statistical Analyses

Body weights, organ weights, adipose tissue deposits, cell counts in IHC, and changes in mRNA and miRNA expressions by qRT-PCR between the control and irradiated mice were analyzed using a Student’s *t*-test. *p* < 0.05 was considered statistically significant. mRNA and miRNA sequencing data were analyzed via DEseq2 methods. The parameters |log2FC| > 0.585 and *p* < 0.05 were considered as significantly differential expression.

## 3. Results

### 3.1. Continuous Prenatal Low-Dose-Rate Irradiation Reduced Organ Weight and Increased Adipose Tissue Deposits in Female B6C3F1 Mice

**Figure 1:**
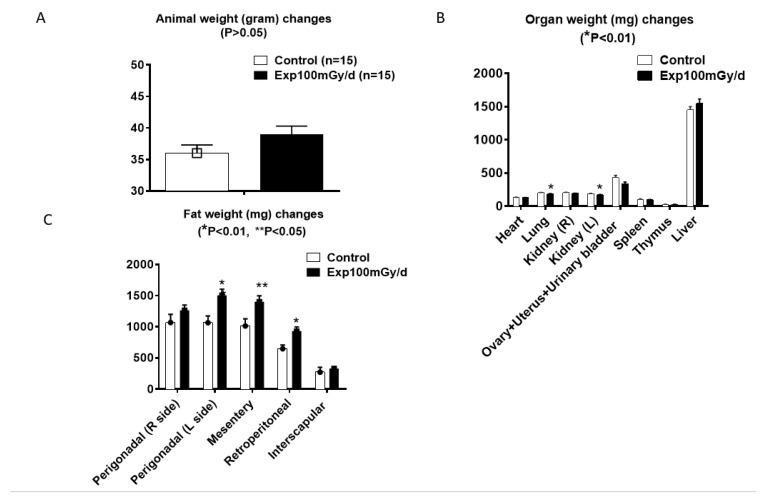
Body weights (**A**) and organ (**B**) and adipose tissue (**C**) weights of female B6C3F1 mice continuously exposed to a low dose rate of 100 mGy/day gamma rays prenatally (gestation day 0–18). n = 15/group. Student’s *t*-test; * *p* < 0.01; ** *p* < 0.05.

Continuous prenatal low-dose-rate irradiation at 100 mGy/day did not significantly affect body weight in female B6C3F1 mice compared to controls (Figure 1A) but reduced the weights of the lungs and left kidney (Figure 1B) and increased adipose tissue deposits in the mesentery, retroperitoneal area, and left perigonadal area (Figure 1C). In contrast, male mice similarly exposed prenatally had lower average body weights and body mass indices and decreased organ weights (heart, liver, testes, epididymides, and kidney) with no significant change in adipose tissue deposits [5].

### 3.2. Immunohistochemistry Examination

**Figure 2:**
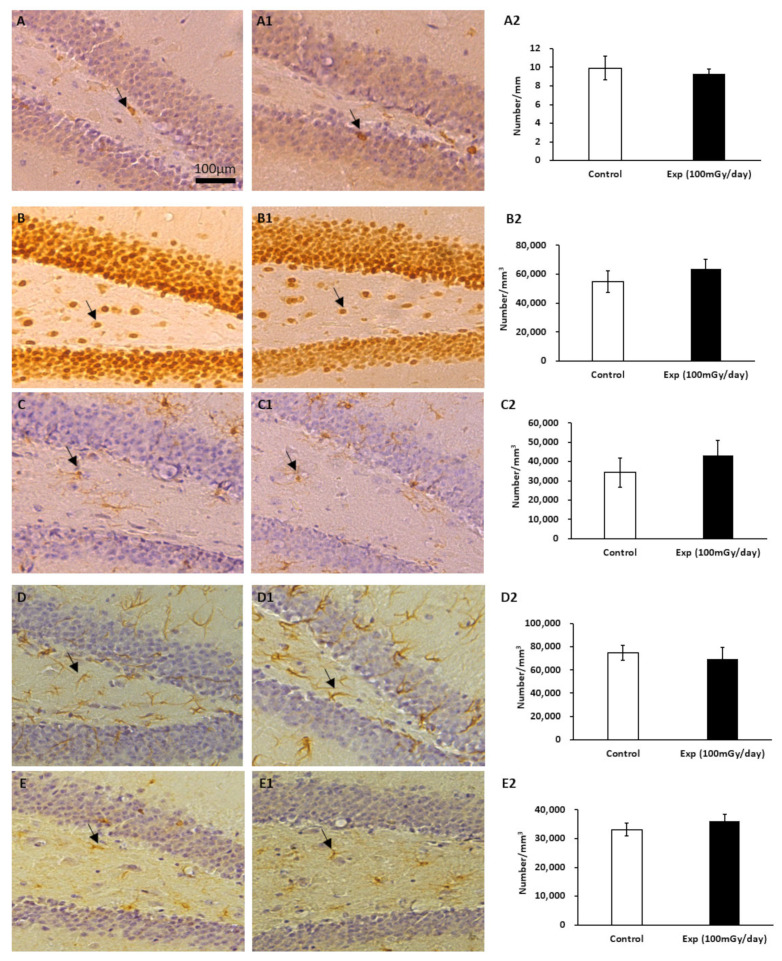
Immunohistochemical staining of the dentate gyrus in the hippocampus of non-irradiated control and irradiated female B6C3F1 mice. Arrows indicate DCX-positive immature neurons in the subgranular zone (**A**,**A1**); NeuN-positive mature neurons (**B**,**B1**); IBA1-positive microglia (**C**,**C1**); GFAP-positive astrocytes (**D**,**D1**); PDGFRα-positive oligodendrocyte precursor cells in the dentate gyrus (**E**,**E1**). Scale bar = 100 μm in (**A**) applies to (**A1**,**B**,**B1**,**C**,**C1**,**D**,**D1**,**E**,**E1**). Statistical results of the positive cell counts for DCX, NeuN, IBA1, GFAP, and PDGFRα were presented in panels (**A2**), (**B2**), (**C2**), (**D2**), and (**E2**), respectively. n = 5/group.

The immunohistochemical analysis did not reveal significant differences in the number of immature neurons (DCX) in the subgranular zone of the dentate gyrus (Figure 2A,A1,A2). Similarly, no changes were observed in the number of mature neurons (NeuN) (Figure 2B,B1,B2), microglia (IBA1) (Figure 2C,C1,C2), astrocytes (GFAP) (Figure 2D,D1,D2), or oligodendrocyte precursor cells (PDGFRα) (Figure 2E,E1,E2) in the dentate gyrus of the hippocampus between the control and irradiated groups.

### 3.3. mRNA and miRNA Sequencing in Female Blood and Hippocampus

**Figure 3:**
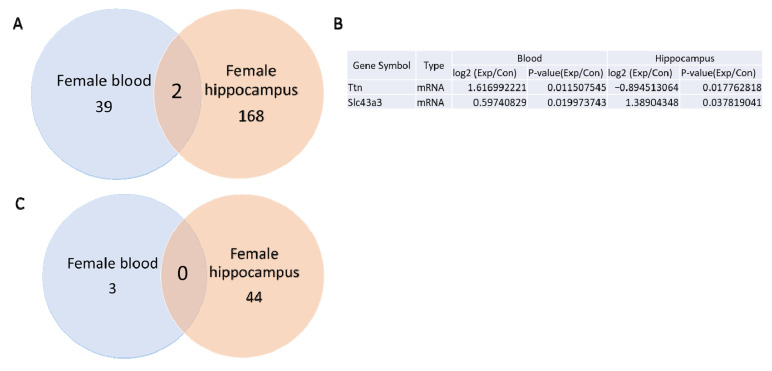
Venn diagrams of mRNA and miRNA sequencing results in the hippocampus and whole blood of the female B6C3F1 mice. (**A**) The Venn diagram shows 2 differentially expressed mRNAs in the hippocampus and whole blood. (**B**) List of differentially expressed mRNAs in whole blood and hippocampus. (**C**) Venn diagram does not show differentially expressed miRNAs between the whole blood and hippocampus.

Using a *p*-value threshold of <0.05 and a fold change > 1.5 between non-irradiated control and irradiated animals, mRNA sequencing analysis identified 41 differentially expressed mRNAs in whole blood (Supplementary Table S1) and 170 in the hippocampus (Supplementary Table S2) of female mice. A Venn diagram analysis (Figure 3A,B) revealed two mRNAs, *Ttn* and *Slc43a3*, were differentially expressed in both whole blood and hippocampal tissues. Notably, *Slc43a3* expression increased in both tissues following gamma irradiation, whereas *Ttn* expression showed an opposite trend, increasing in the entire blood but decreasing in the hippocampus.

No miRNAs were found to be differentially expressed in both whole blood and hippocampus (Figure 3C). Altered expressions of 3 miRNAs in the blood (Supplementary Table S3) and 44 miRNAs in the hippocampus (Supplementary Table S4) were observed.

### 3.4. qRT-PCR Validation of mRNA and miRNA Expression in Female Hippocampus and Blood

**Figure 4:**
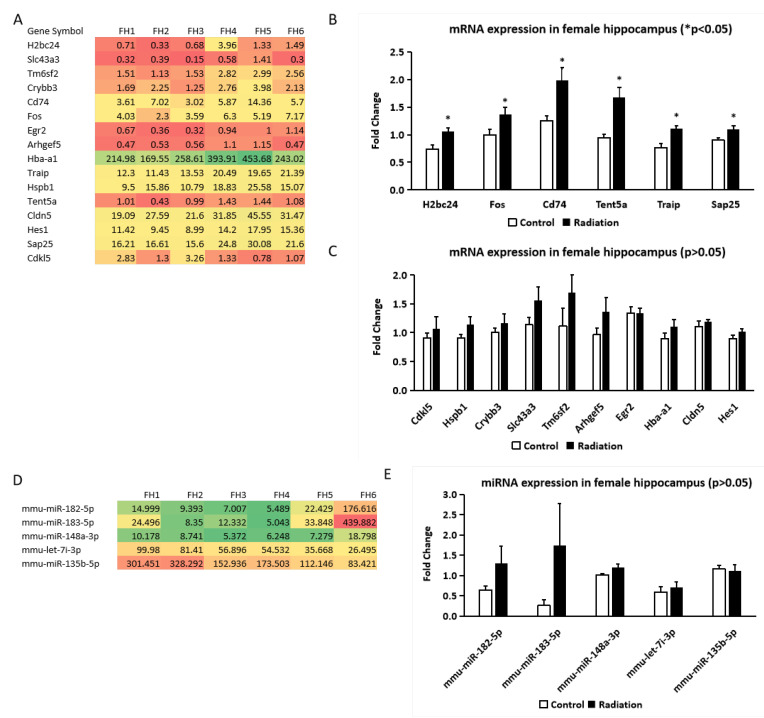
Differentially expressed mRNAs and miRNAs in the hippocampi of non-irradiated control and prenatally irradiated female B6C3F1 mice. (**A**) Heatmap of mRNAs from mRNA sequencing data; (**B**,**C**) qRT-PCR results of the selected mRNAs; (**D**) heatmap of miRNAs from miRNA sequencing data; (**E**) qRT-PCR results of these selected. (**A**,**D**): |log2FC| > 0.585 and *p* < 0.05; (**B**,**C**,**E**): Student’s *t*-test, * *p* < 0.05. FH1–3: n = 3 non-irradiated control and FH4–6: n = 3 irradiated mice.

For qRT-PCR analysis, 16 mRNAs were selected based on the Venn diagram comparisons between whole blood and hippocampus in the female mice, as well as between female and male whole blood and hippocampus. These genes, related to neurogenesis and fat deposition, were highlighted in a heatmap (Figure 4A). qRT-PCR confirmed the upregulation of *H2bc24*, *Fos*, *Cd74*, *Tent5a*, *Traip*, and *Sap25* in the female hippocampus following gamma irradiation (Figure 4B). However, the expression levels of 10 other mRNAs (*Slc43a3*, *Tm6sf2*, *Crybb3*, *Egr2*, *Arhgef5*, *Hba-a1*, *Hspb1*, *Cldn5*, *Hes1*, and *Cdkl5*) did not show any significant change after irradiation (Figure 4C).

miRNA sequencing identified five differentially expressed miRNAs (*mmu-miR-182-5p*, *mmu-miR-183-5p*, *mmu-miR-148a-3p*, *mmu-let-7i-3p*, and *mmu-miR-135b-5p*) in the hippocampus (Figure 4D) but was not validated by qRT-PCR (Figure 4E).

**Figure 5:**
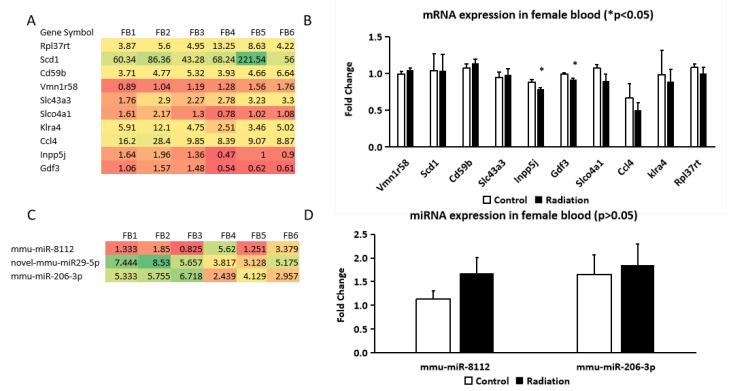
Differentially expressed mRNAs and miRNAs in whole blood of the non-irradiated control and prenatally irradiated female B6C3F1 mice. (**A**) Heatmap of mRNAs in whole blood from mRNA sequencing data; (**B**) qRT-PCR indicates a down-regulation of Inpp5j and Gdf3 in whole blood; (**C**) Heatmap of miRNAs in whole blood from miRNA sequencing data; (**D**) qRT-PCR shows no difference in the expression of the selected miRNAs in whole blood. (**A**,**C**): |log2FC| > 0.585 and *p* < 0.05; (**B**,**D**): Student’s *t*-test, * *p* < 0.05. FB1–3: n = 3 non-irradiated control mice (FB); FB4–6: n = 3, irradiated mice.

Among the 10 selected mRNAs that were differentially expressed in whole blood after gamma irradiation (Figure 5A), only two genes, *Inpp5j* and *Gdf3*, were downregulated according to the qRT-PCR assay (Figure 5B). The qRT-PCR analysis did not confirm significant changes in expression for the remaining eight genes (*Rpl37rt*, *Scd1*, *Cd59b*, *Vmn1r58*, *Slc43a3*, *Slco4a1*, *Klra4*, and *Ccl4*) (Figure 5B).

Similarly, among the three differentially expressed miRNAs identified by miRNA sequencing in whole blood (Figure 5C), the qRT-PCR showed no significant differences for two miRNAs (*mmu-miR-8112* and *mmu-miR-206-3p*) (Figure 5D).

### 3.5. Comparison of mRNA and miRNA Sequencing of Whole Blood from Prenatally Irradiated Male and Female B6C3F1 Mice

**Figure 6:**
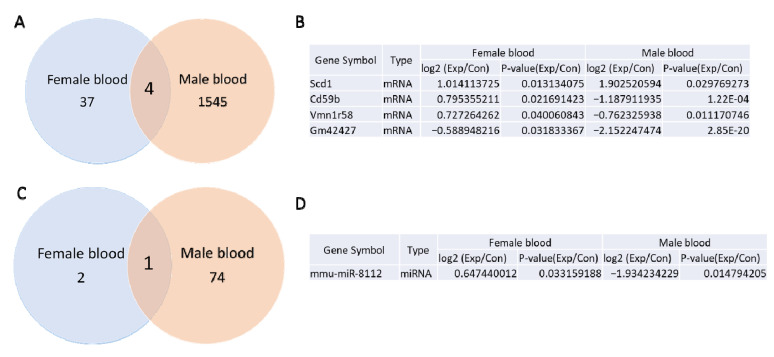
mRNA and miRNAs sequencing results in prenatally irradiated female and male B6C3F1 whole blood samples**.** Venn diagram shows 4 differentially expressed mRNAs (**A**) between female and male, which are tabulated in (**B**). Venn diagram shows 1 differentially expressed miRNA between female and male (**C**), which is in (**D**).

Based on a *p*-value of less than 0.05 and a fold change greater than 1.5 between non-irradiated control and irradiated samples, mRNA sequencing analysis identified 41 and 1549 differentially expressed mRNAs in whole blood of irradiated female and male mice, respectively [5]. Venn diagram analysis revealed four mRNAs that were differentially expressed in both female and male whole blood samples of prenatally irradiated mice. Among these, Scd1 expression increased, while Gm42427 expression decreased in both groups following gamma irradiation. In contrast, Cd59b and Vmn1r58 exhibited opposite expression patterns: their levels increased in females but decreased in males (Figure 6A,B).

Gamma irradiation also induced differential expression of 3 miRNAs in female whole blood (Supplementary Table S3) and 75 miRNAs in male whole blood [5]. Notably, mmu-miR-8112 was differentially expressed in the whole blood of both males and females, increasing in females but decreasing in males after irradiation (Figure 6C,D).

### 3.6. Comparison Between mRNA and miRNA Sequencing of the Hippocampus from Prenatally Irradiated Male and Female B6C3F1 Mice

**Figure 7:**
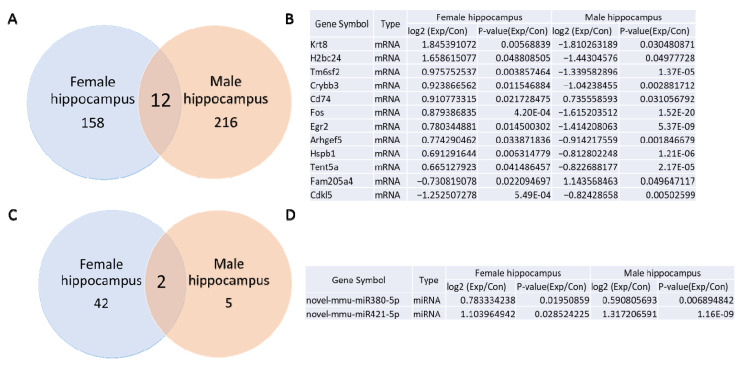
Venn diagram of mRNA and miRNAs sequencing results in female and male hippocampus**.** (**A**). Venn diagram indicates 12 differentially expressed mRNAs between female and male hippocampus in B6C3F1 mice; (**B**). Table listing of 12 differentially expressed mRNAs in female and male hippocampus. (**C**). Venn diagram indicates 2 differentially expressed miRNAs between female and male hippocampus in B6C3F1 mice; (**D**). Table listing of 2 differentially expressed miRNAs between female and male hippocampus.

Based on a *p*-value of less than 0.05 and a fold change greater than 1.5 between the control and irradiated samples, the mRNA sequencing analysis identified 170 (Supplementary Table S2) or 228 [5] differentially expressed mRNAs in the female and male hippocampus of irradiated mice compared to controls. The Venn diagram analysis revealed 12 mRNAs that were differentially expressed in both female and male hippocampal samples (Figure 7A,B). Among these, Cd74 expression increased after gamma irradiation in both groups, while Cdkl5 expression decreased (Figure 7B). Additionally, Krt8, H2bc24, Tm6sf2, Crybb3, Fos, Egr2, Arhgef5, Hspb1, and Tent5a showed opposite expression patterns: their levels increased in the female hippocampus but decreased in the male hippocampus. In contrast, Fam205a4 expression decreased in the female hippocampus and increased in the male hippocampus.

The miRNA sequencing analysis revealed 44 (Supplementary Table S4) or 7 differentially expressed miRNAs [5] in the female and male hippocampus following gamma irradiation. The Venn diagram analysis indicated that two novel miRNAs—novel-mmu-miR380-5p and novel-mmu-miR421-5p—were upregulated in both female and male hippocampal samples.

## 4. Discussion

### 4.1. Prenatal Continuous Low-Dose-Rate Irradiation Induced mRNA and miRNA Changes in Whole Blood and the Hippocampus, with No Cellular Changes in the Dentate Gyrus of Female Offspring

In this study, we examined the effects of prenatal continuous low-dose rate (100 mGy/day from gestation day 0–18 to a total dose of 1.8 Gy) gamma-ray exposure on the hippocampal structure and gene expression in female offspring. Notably, no cellular changes were observed in the dentate gyrus, including the absence of mature neuronal loss, reactive glial response in the hilus (area susceptible to brain insults), or impaired neurogenesis in the subgranular zone. These results are similar to our previous study on similarly exposed male offspring [5].

Despite the absence of histological changes in the hippocampus, mRNA and miRNA sequencing revealed significant alterations in gene expressions. In the hippocampus, 170 mRNAs and 44 miRNAs showed changes, while 41 mRNAs and 3 miRNAs were altered in whole blood. Among the mRNAs, Slc43a3 and Ttn were notably altered in both the hippocampus and whole blood where Slc43a3 was upregulated in both, while Ttn expression increased in whole blood but decreased in the hippocampus. These altered expressions, however, were not confirmed by qRT-PCR analysis in either the hippocampus or blood.

qRT-PCR validated the upregulation of H2bc24, Fos, Cd74, Tent5a, Traip, and Sap25 genes in the hippocampus, and the downregulation of Inpp5j and Gdf3 in whole blood. Although the specific roles of these gene changes remain unclear, partly due to the animals being euthanized at a single end point (1 year of age), several speculative insights can be drawn: H2bc24, Fos, Cd74, Tent5a, Traip, and Sap25 may contribute to maintaining hippocampal genome stability, transcriptional activity, neuroinflammation, glioma progression, and immune responses at later stages of animal life [19,20,21,22,23]. The downregulation of Gdf3 and Inpp5j in whole blood might represent a self-protective mechanism against adipogenesis and carcinogenesis, as elevated levels of Gdf3 and Inpp5j are associated with fat deposition [24] and cancer development [25], respectively.

These findings suggest that while prenatal low-dose irradiation does not cause overt structural damage in the hippocampus, it induces complex molecular changes that could have long-term functional implications. Future studies at multiple time points will be crucial to fully understand the roles of these genetic alterations in aging and disease susceptibility.

### 4.2. Sex Differences in Weight Changes, Adipose Tissue Deposit, and Expression of mRNA and miRNA After Continuous Prenatal Low-Dose-Rate Irradiation

#### 4.2.1. Weight Changes and Lipid Metabolism

We observed a sex-specific change in body weight where male B6C3F1 mice exhibited reduced body weights and BMIs after the prenatal continuous low-dose rate gamma irradiation [5], while no significant change was observed in female B6C3F1 mice. These findings align with a study which demonstrated that radiation exposure negatively affected the BMI in men but had no impact on women [26]. In men, increasing radiation doses were associated with reduced BMI and cholesterol levels. Lipogenesis, the process of synthesizing fatty acids from glucose and converting them into triglycerides, primarily occurs in the cytoplasm and endoplasmic reticulum of the liver and adipose cells. Fat accumulation is regulated by the balance between synthesis and lipolysis/fatty acid oxidation and is highly responsive to radiation-induced changes. In this study, we observed increased adipose tissue deposition in the mesentery, retroperitoneal region, and left perigonadal area in female mice following low-dose gamma irradiation, accompanied by reduced lung and left kidney weights. In contrast, our previous research showed that prenatal irradiation at 100 mGy/day for 18 days did not induce changes in adipose tissue deposition in male mice, although organ weight decreases were noted in the heart, liver, and kidneys [5]. Further study is still needed to indicate if prenatal and postnatal, high- and low-dose rate, and acute and chronic irradiation will produce similar effects on male and female animals and human beings.

#### 4.2.2. mRNA and miRNA Expression

Sex differences in radiation response have been well documented [6,7,8,9,10,11], and males are, in general, more vulnerable than their female counterparts to, for example, cognitive impairments [27], although increased radiosensitivity in females is detectable only in the mammillary bodies and fornix [28]. Galactic cosmic ray simulation affected plasma IL-2, IL-5, IL-6, and IL-10 in males, but keratinocyte chemoattractant (KC)/human growth-regulated oncogene (GRO) (KC/GRO) was the only cytokine altered in female [27]. In the brain, the difference in mRNA and miRNA expression patterns between male and female mice has been investigated extensively in the radiation exposure models. The miRNA expression basal levels in the female and male control frontal cortex, hippocampus, and cerebellum had a stable tissue- and sex-specific miRNA expression pattern. Radiation-induced miRNA changes were less pronounced in male than female mice. While both miR-34c and miR-488* were altered after irradiation, common miRNA dysregulation was not detected in hippocampus between male and female mice or among the time points after acute irradiation with 1 Gy (3 Gy/h) [29].

The expression of Fos has been implicated in neuronal synaptic plasticity and memory formation [30]. In radiation-induced brain injury in adult male rats, a single high dose of 20 Gy to the whole brains of rats increased c-Fos gene expression levels in the hippocampus [31]. Fos-like immunoreactive cells were also induced in the cerebral cortex and the hippocampus after gamma irradiation (100 Gy) of the forebrain in the male rat [32]. In male NMRI mice, a marked decrease in the gene and protein expression of c-Fos in the hippocampus and cortex was reported after a total body gamma irradiation with 1.0 Gy on postnatal day 10 [33]. In male C57BL/6 mice, the whole-brain X-ray irradiation with 10 Gy (dose rate of 2.0 Gy/min) upregulated Fos gene expression in the hippocampus and the cortex [34]. While our previous chronic prenatal low-dose-rate irradiation did not induce c-Fos gene upregulation but downregulation in the hippocampus of male mice [5], the present study did show the upregulation of c-Fos expression in female mice, suggesting a sex difference in c-Fos gene expression after the prenatal low-dose-rate irradiation. The importance of a potential long-term global elevation of c-Fos in the hippocampus remains unknown. Lee et al. (2022) speculated that upregulation of c-fos gene at the chronic stage after irradiation may be implicated in neuronal plasticity and memory formation in the brain [34]. In male mice, the downregulation of c-Fos gene expression suggests the existence of difference in radiation responses between either prenatal and postnatal irradiation or between acute high-dose-rate and chronic low-dose-rate radiation exposure. Downregulation of c-Fos gene and protein exposures after the acute irradiation (1 Gy) of postnatal-day-10 male NMRI mice suggests that animal age (mature vs. immature), species (rat vs. mouse), strain (C57BL/6 vs. NMRI mice), radiation dose (1 vs. 10), irradiation pattern (whole brain vs. whole body), or animal condition (anesthetized or aware) may also affect their radiation responses.

The expression of Fos and Tent5a increased in the hippocampus of female subjects but decreased in males. Limited information is available regarding the roles of Tent5a in radiation research. Radiation-induced upregulation of Cd74 has been observed in residual cervical cancer tissues after fractionated radiotherapy with 50 Gy [35] and in the old mouse spleen after whole body fractionated irradiation with 0.5 Gy of proton ions per day for 3 days [36]. CD74 may be implicated in lipid metabolism. When six-week-old C57BL/6J male mice and CD74 knockout male mice were fed a diet supplemented with 30% fat, total liver triglyceride (TG) levels significantly increased in C57BL/6J mice but not in the CD74 knockout group [37]. Furthermore, CD74 inhibition has been shown to reduce inflammation in adipose tissue and mitigate insulin resistance associated with high-fat-diet-induced obesity [38]. CD74 was also significantly upregulated at both transcript and protein levels in Bama miniature pigs fed a high-fat, high-sucrose diet [39]. As the hippocampus plays a role in regulating metabolism through its connections with the hypothalamus and other brain regions, from a speculative point of view, the above evidence suggests that prenatal low-dose-rate-radiation-induced elevated Cd74 expression in the hippocampus in female B6C3F1 mice may be related to increased adipose tissue deposits in the mesentery, retroperitoneal area, and left perigonadal area.

In female blood, among the 10 mRNAs tested, the expression of two genes—Inpp5j and Gdf3—was downregulated according to both sequencing and qRT-PCR results. INPP5J proteins are enzymes that dephosphorylate the 5-phosphate position of phosphoinositides (PI). Mutations or misexpression of PI phosphatases, including INPP5J, are associated with several human genetic diseases, such as Lowe syndrome, developmental disorders, and cancers, often leading to loss of function [40,41,42,43]. However, limited information is available regarding their function in radiation research. It remains to be investigated whether the downregulation of Inpp5j is involved in the development of any diseases, such as cancer, at the late stages of animal life.

Growth differentiation factor 3 (GDF3), a member of the TGF-beta superfamily, is highly expressed in white adipose tissue [24,44,45]. Studies have shown that GDF3 regulates adipose tissue accumulation and high-fat-diet-induced obesity through ALK7-signaling pathways [45]. Given ALK7′s pivotal role in lipid metabolism and fat mass regulation, the GDF3-ALK7 signaling axis is considered a potential therapeutic target for obesity and associated diabetes [45]. In the present study, the increased adipose tissue deposits in the mesentery, retroperitoneal area, and left perigonadal area but not in the right perigonadal area and interscapular region suggest that radiation-induced increased blood Gdf3 or GDF3-ALK7 signaling may be involved in adipose tissue deposition in the former three regions.

In contrast to the differential expression observed for mRNAs in whole blood and the hippocampus, miRNA-sequencing data did not reveal differential expression of common miRNAs in both tissues. Sequencing, however, detected 3 differentially expressed miRNAs (mmu-miR-8112, novel-mmu-miR29-5p, and mmu-miR-206-3p) in whole blood and changes in 44 miRNAs, including mmu-miR-182-5p, mmu-miR-183-5p, mmu-miR-148a-3p, mmu-let-7i-3p, and mmu-miR-135b-5p, in the hippocampus. The qRT-PCR was unable to validate these selected miRNA changes. Further studies with increased sample sizes or qRT-PCR validation of other miRNAs may be needed.

The link between radiation exposure and lipid metabolism may involve changes in gonadal white adipose tissue deposits and the expression of lipogenesis-related genes. Our mRNA and miRNA sequencing detected differential expression of 41 mRNAs and 3 miRNAs in whole blood and 170 mRNAs and 44 miRNAs in the hippocampus of female mice exposed to prenatal irradiation. Comparative analysis using Venn diagrams identified two overlapping mRNAs (Ttn and Slc43a3) in the blood and hippocampus; Slc43a3, a regulator of free fatty acid flux, is highly expressed in adipose tissue and produced during adipocyte differentiation [46]. Its dose-dependent expression relationship has been implicated in papillary thyroid cancer cases from the Chernobyl incident [47]. Our qRT-PCR study did not validate Ttn and Slc43a3 changes from mRNA sequencing analysis in both blood and hippocampus. This may be due to a small sample size or other unknown factors.

## 5. Conclusions

The continuous prenatal low-dose-rate (100 mGy/day) gamma irradiation with a cumulative dose of 1.8 Gy resulted in an increased adipose tissue deposits in the mesentery, retroperitoneal, and left perigonadal areas and reduced lung and kidney weights with no significant change in the body weight of female but not male B6C3F1 mice [5]. Similar to their male counterparts, these female mice also did not show cellular changes in immature neurons in the subgranular zone or mature neurons and glial cells, including astrocytes, microglia, and oligodendrocyte progenitor cells, in the hilus of the dentate gyrus. The irradiation induced the upregulation of mRNAs such as H2bc24, Fos, Cd74, Tent5a, Traip, and Sap25 in the hippocampus, and the downregulation of Inpp5j and Gdf3 in the whole blood of female mice. A similar upregulation of Cd74 and downregulation of Fos and Tent5a occurred in the hippocampus of male mice similarly exposed. Further study at the late stages of animal life is needed to investigate the functional roles of these changes in gene expression in the hippocampus and whole blood.

## Figures and Tables

**Table 1:** PCR primer sequence for female hippocampus mRNAs.

Gene Name	Primer Sequence
*H2bc24* F	AGAGTTCCAGAGTTCCAGTCTCATC
*H2bc24* R	GAACTCACTTGGAGCTGGTGT
*Slc43a3* F	CCTCACGCTGATTTCCCTCA
*Slc43a3* R	AGGAGACATTGCTCACAGGC
*Tm6sf2* F	TTCTCACACATGGGTGCCTC
*Tm6sf2* R	CTTGGTCCTGTGGCGAAGAT
*Crybb3* F	AAGCAGGTCTCTGCCTCCT
*Crybb3* R	TACGATCTCCATCTTGCGCC
*Cd74* F	AGTGCCAGGAAGAAGTCAGC
*Cd74* R	CCAGCGTCCTCCTTCTGTTC
*Fos* F	AGTCAAGGCCTGGTCTGTGT
*Fos* R	TGGAACACGCTATTGCCAGG
*Hba-a2* F	GCTGAAGCCCTGGAAAGGAT
*Hba-a2* R	GGAGCTTGAAGTTGACGGGA
*Egr2* F	GCCAGGAGTGACGAAAGGAA
*Egr2* R	GTGAGAAGGTGGGACAGAGC
*Arhgef5* F	GACTCTGGGTGGTCGTGGAG
*Arhgef5* R	GGCCTCAGCCAGAAGGATTT
*Hba-a1* F	GCTGAAGCCCTGGAAAGGAT
*Hba-a1* R	GGGAGAGAAGAAGGGCATGG
*Traip* F	ACCTTTTGACCCTGTTGGTGT
*Traip* R	GTAAGCAGGCCTCCTGAGTG
*Hspb1* F	ATAGAGACCTGAAGCACCGC
*Hspb1* R	CGGTCATGTTCTTGGCTGGT
*Tent5a* F	CTCCAGGACTGACCAAGGC
*Tent5a* R	CGGACACCTATGCCCTTCTC
*Cldn5* F	GCTCTCAGAGTCCGTTGACC
*Cldn5* R	TTCTCCAGCTGCCCTTTCAG
*Hes1* F	GCCGTCTATCCGTATTGCCA
*Hes1* R	GTTTGTCCGGTGTCGTGTTG
*Sap25* F	GTTGTGGGCGCTTTCCAAAA
*Sap25* R	CGAAGTGGCAGTGGAGACAT
*Cdkl5* F	AACGGCGAGAATCCAAGCAT
*Cdkl5* R	AAGGCGTTTGTTGGTCACTGT
*GAPDH* F	ACCACAGTCCATGCCATCAC
*GAPDH* R	TCCACCACCCTGTTGCTGTA

**Table 2:** PCR primer sequence for female blood mRNAs.

Gene Name	Primer Sequence
*Rpl37rt* F	CCAAGGCCTACCACCTTCAG
*Rpl37rt* R	AAGAACTGGATGCTGCGACA
*Scd1* F	GAGTAGCTGAGCTTTGGGCT
*Scd1* R	ACTTCATCAGCGGGGACTTG
*Cd59b* F	CTGTTGCCTTGGATCAGCCT
*Cd59b* R	TGATACACTTGCCTTCCGGC
*Vmn1r58* F	GGTCAAAACACGGCCAAACC
*Vmn1r58* R	AGGAGAAACAGCCTTCTCTCAA
*Slc43a3* F	CCTCACGCTGATTTCCCTCA
*Slc43a3* R	AGGAGACATTGCTCACAGGC
*Slco4a1* F	CTTGGGCGATGAATGAAGCG
Slco4a1 R	ACACATACTGCACCTCACGG
*Klra4* F	CGCCTCAGAGTGTGTTCAGT
*Klra4* R	TGTCTGAAGGAACCACGAGC
*Ccl4* F	CTAACCCCGAGCAACACCAT
*Ccl4* R	TGAACGTGAGGAGCAAGGAC
*Inpp5j* F	ATCTGCCACTCTGTCTTGGC
*Inpp5j* R	TCTGTCACATCTGCAACTGCT
*Gdf3* F	GTGCCCCTTCTCAATGACCA
*Gdf3* R	GCTCACCAAGGGGTCCATAG
*GAPDH* F	ACCACAGTCCATGCCATCAC
*GAPDH* R	TCCACCACCCTGTTGCTGTA

**Table 3:** PCR primer sequence for female hippocampus and blood miRNAs.

miRNA	Primer Sequence
mmu-miR-182-5p	TTTGGCAATGGTAGAACTCACACCG
mmu-miR-183-5p	TATGGCACTGGTAGAATTCACT
mmu-miR-148a-3p	TCAGTGCACTACAGAACTTTGT
mmu-let-7i-3p	CTGCGCAAGCTACTGCCTTGCT
mmu-miR-135b-5p	TATGGCTTTTCATTCCTATGTGA
mmu-miR-8112	ATATCTCCGCCACCTCCACCGCA
mmu-miR-206-3p	TGGAATGTAAGGAAGTGTGTGG
mmu-miR-68	GCTGTACTGACTTGATGAAAGTAC

## Data Availability

The data presented in this study are available on request from the corresponding author.

## Supplementary Materials

The following supporting information can be downloaded at https://www.mdpi.com/article/10.3390/cells14030173/s1: Table S1. Heatmap of mRNA-sequencing data in female blood; Table S2. Heatmap of mRNA-sequencing data in female hippocampus; Table S3. Heatmap of miRNA-sequencing data in female blood; Table S4. Heatmap of miRNA-sequencing data in female hippocampus.

## Abbreviations

ALK7	Activin receptor-like kinase 7
Arhgef5	Rho guanine nucleotide exchange factor 5
Ccl4	Chemokine (C-C motif) ligands 4
Cdkl5	Cyclin dependent kinase like 5
Cldn5	Claudin 5
Crybb3	Crystallin Beta B3
DCX	Doublecortin
Egr2	Early growth response protein 2
Fos	Fos proto-oncogene
GAPDH	Glyceraldehyde-3-phosphate dehydrogenase
Gdf3	Growth differentiation factor 3
GFAP	Glial fibrillary acidic protein
H2bc24	H2B clustered histone 24
Hba-a1	Hemoglobin Subunit Alpha 1
Hba-a2	Hemoglobin subunit alpha 2
HDAC	Histone deacetylase
Hes1	Hairy and enhancer of split-1
Hspb1	Heat shock protein family B (small) member 1
Iba1	Ionized calcium binding adapter protein 1
Inpp5j	Inositol polyphosphate-5-phosphatase J
Klra4	Killer cell lectin-like receptor, subfamily A, member 4
NeuN	Neuronal nuclear protein
PDGFRA	Platelet derived growth factor receptor alpha
Rpl37rt	Ribosomal protein L37, retrotransposed
Sap25	Sin3A associated protein 25
Scd1	Stearoyl-CoA desaturase 1
Sin3A	SIN3 Transcription Regulator Family Member A
Slc43a3	Solute carrier family 43 member 3
Slco4a1	Solute carrier organic anion transporter family member 4A1
Tent5a	Terminal nucleotidyltransferase 5A
Tm6sf2	Transmembrane 6 superfamily member 2
Traip	TRAF interacting protein
Ttn	Titin
Vmn1r58	Vomeronasal 1 receptor 58
