# Supplementary material for: Alterations in Blood and Hippocampal mRNA and miRNA Expression, Along with Fat Deposition in Female B6C3F1 Mice Continuously Exposed to Prenatal Low-Dose-Rate Radiation and Their Comparison with Male Mice
Source: Cells. 2025 Jan 23;14(3):173. doi: 10.3390/cells14030173 (PMC11816924; doi:10.3390/cells14030173)
Supplement: Supplementary file 1 [file cells-14-00173-s001.zip › cells-3438732-supplementary.pdf]

Table S1. Heatmap of mRNA sequencing data in female blood

Gene Symbol	FB1	FB2	FB3	FB4	FB5	FB6
Fam166a	0.06	0	0	0	1.54	0.06
2010003K11Rik	0.06	0.23	0	0.22	4	0.17
Fscn2	0.02	0	0	0.1	0.14	0.18
Gm20385	0.01	0	0.03	0.07	0.13	0.05
Mup7	0	0.87	0	0.22	6.6	0.83
Gm38574	0	0	0.28	1.04	0.46	0.81
G6pc	0.34	0.63	0	0.8	8.06	0.24
Fitm1	0.08	1.09	0	1.31	7.05	0.45
Mup20	0.19	0.85	0.08	0.53	6.05	0.09
H3c13	0.41	0.32	0	0.99	1.31	0.87
Rpl17-ps8	0	0.16	0.24	0.85	0.61	0.44
Rybp-ps	0.06	0	0.06	0.17	0.2	0.12
Ltf	0.49	3.05	1.12	8.77	1.03	1.91
Gm40453	1.96	2.88	5.6	5.62	14.01	11.3
Ttn	0.01	0.01	0.01	0.03	0.02	0.04
Gm15682	14.52	14.29	51.97	69.12	93.82	74.76
Rpl37rt	3.87	5.6	4.95	13.25	8.63	4.22
Scd1	60.34	86.36	43.28	68.24	221.54	56
Bhlha15	0.25	0.31	0.31	0.49	0.58	0.39
Gm41844	6.05	5.09	9.32	7.9	13.19	12.36
Slc43a1	2.77	2.9	3.14	5.88	3.97	3.48
Cd59b	3.71	4.77	5.32	3.93	4.66	6.64
Vmn1r58	0.89	1.04	1.19	1.28	1.56	1.76
Slc43a3	1.76	2.9	2.27	2.78	3.23	3.3
Gm42427	4.46	9.41	5.58	3.54	3.7	3.8
Slco4a1	1.61	2.17	1.3	0.78	1.02	1.08
Gm14325	2.24	2.12	1.39	0.71	1.64	1.06
LOC118567871	0.6	0.68	0.37	0.22	0.43	0.29
Zc3h12c	0.43	0.71	0.41	0.19	0.38	0.25
Klra4	5.91	12.1	4.75	2.51	3.46	5.02
Gm45855	30.29	27.58	14.07	11.44	12	12.62
Ccl4	16.2	28.4	9.85	8.39	9.07	8.87
Inpp5j	1.64	1.96	1.36	0.47	1	0.9
LOC118568310	7.04	4.05	3.84	2.67	1.84	2.37
Fras1	0.28	0.51	0.58	0.19	0.31	0.17
Gdf3	1.06	1.57	1.48	0.54	0.62	0.61
Klra1	1.26	1.84	1.09	0.25	0.85	0.66
Gm45988	19.39	18.63	8.58	2.45	3.23	5.37
Grip2	0.03	0.06	0.06	0	0	0
Gm46142	3.91	2.18	0	0.24	0	0.09
Cyp3a16	0.11	14.89	0	0.04	0.21	0.15

Table S2. Heatmap of mRNA sequencing data in female hippocampus

Gene Symbol	FH1	FH2	FH3	FH4	FH5	FH6
Krt7	0	0	0	0.1	0.47	0.1
Klhl30	0	0	0	0.09	0.11	0.07
S100a5	0.34	0	0	0.34	0.19	3.77
Rab19	0	0	0	0.11	0.33	0.06
Mcoln3	0	0	0.05	0.05	0.5	0
Serpinb6c	0	0.07	0	0.13	0.63	0.13
H2-Q7	0	0	0.12	0.55	0.16	0.32
Tmem102	0	0.08	0.04	0.54	0.45	0.15
Cldn9	0	0.05	0.11	0.54	0.95	0.11
Epcam	0.21	0.07	0.03	0.07	2.82	0.25
Otoa	0	0.06	0.02	0.08	0.56	0.02
Myoz1	0	0	0.12	0.43	0.3	0.31
Cldn3	0.32	0.13	0.06	1.47	2.35	0.45

Slc38a8	0.03	0.03	0	0.11	0.22	0.11
Steap4	0.09	0.02	0.07	0.54	0.37	0.02
Timp1	0	0.5	0	0.71	1.4	0.64
Omp	0.21	0.25	0.21	0.39	0.45	2.38
Large2	0.06	0.12	0.3	1.22	0.81	0.16
Nrp	0.15	0.21	0.07	0.22	1.33	0.36
Il17re	0.06	0.03	0.32	0.77	0.88	0.09
Wfdc2	0.56	0.42	2.06	6.29	5.27	0.28
Slc47a1	0.19	0.65	0.85	1.41	3.85	1.18
Efhb	0.16	0.06	0.05	0.4	0.42	0.19
Ptgis	0.35	0.41	0.72	2.07	2.76	0.75
Krt8	0.41	0.25	0.65	1.92	2.59	0.34
D630039A03Rik	0.07	0.12	0.12	0.47	0.55	0.12
Smim5	0.41	0.19	0.5	0.9	2.95	0.5
H2bc24	0.71	0.33	0.68	3.96	1.33	1.49
Pifo	0.97	0.49	0.96	2.45	4.26	0.79
Mdfic	0.41	0.57	1.09	2.53	3.12	0.7
Depp1	0.72	0.6	1.3	2.43	2.65	2.93
Mpzl2	0.09	0.42	0.34	0.51	1.6	0.35
Rhod	0.4	0.34	0.51	1.17	1.38	0.4
Slc43a3	0.32	0.39	0.15	0.58	1.41	0.3
Llgl2	0.24	0.35	0.5	0.99	1.48	0.38
Fut7	0.26	0.39	0.17	0.54	0.69	0.91
Gpx8	2.49	3.18	5.33	13.08	11.05	3.13
Sphk1	0.47	1.4	1.01	1.42	4.48	1.43
Thbs1	0.21	0.2	0.46	0.71	1.11	0.29
Tgtp1	0.25	0.67	0.85	1.99	1.8	0.36
Dnah11	0.48	0.46	0.5	1.12	2.11	0.31
Mapk15	1.33	1.22	0.93	2.18	4.32	1.58
Dynlrb2	3.31	2.81	2.36	5.7	9.63	3.35
Gstm2	1.56	2.22	3.46	5.12	9.36	1.58
Tjp3	0.63	0.46	0.69	1.93	1.48	0.39
H2-Ab1	0.89	1.57	1.2	2.13	3.99	1.72
Col18a1	0.81	0.89	1.53	2.98	2.82	1.12
Hhipl2	0.56	0.37	0.32	0.78	1.03	0.85
Cdkn1c	2.7	3.27	3.68	8.49	9.79	2.19
Crb3	0.92	1.43	1.4	2.92	3.26	1.37
Il1f9	1.18	1.29	0.61	1.07	3.36	1.86
H2-Aa	1.53	3.37	2.01	4.27	7.56	2.52
Bmp5	0.22	0.33	0.35	0.55	0.83	0.46
Cfap126	2.93	2.99	2.11	6.01	8.15	2.17
Tm6sf2	1.51	1.13	1.53	2.82	2.99	2.56
Elmo3	0.42	0.68	0.54	1.11	1.42	0.44
Nid2	0.54	0.7	1.31	2.52	1.66	0.79
Crybb3	1.69	2.25	1.25	2.76	3.98	2.13
Acss3	0.41	0.37	0.49	0.75	1.26	0.46
Crybg1	0.13	0.22	0.17	0.32	0.34	0.33
Cd74	3.61	7.02	3.02	5.87	14.36	5.7
Bst2	3.66	4.68	4.39	10.36	9.02	4.86
Zfp990	0.82	0.68	0.67	1.87	1.33	0.9
Fos	4.03	2.3	3.59	6.3	5.19	7.17
Rbp1	2.58	2.97	3.6	6.82	7.56	2.72
Itpripl1	0.53	0.62	0.94	1.54	1.46	0.92
Sfrp1	1.54	1.32	2.06	4.19	3.4	1.46
Col14a1	0.31	0.19	0.26	0.44	0.61	0.34
St6galnac2	2.28	3.85	5.02	7.33	9.61	3.3
Emilin1	0.52	0.68	0.53	1.03	1.4	0.69
Hba-a2	44.63	43	51.63	96.09	106.18	46.28
Col6a2	6.16	5.14	5.44	6.38	13.57	10.41
Ltc4s	6.03	6.77	9.56	16.02	17.86	6.26
Atoh8	1.35	1.29	0.99	1.76	3.06	1.65
Spp1	1.92	3.36	3.77	4.27	8.18	3.62

Capsl	2.67	3.77	2.98	5.91	7.29	3.79
Itga10	0.69	0.84	1.03	1.52	1.56	1.42
Zfp185	1.16	1.09	1.22	2.64	2.51	0.95
Egr2	0.67	0.36	0.32	0.94	1	1.14
Arhgef5	0.47	0.53	0.56	1.1	1.15	0.47
Foxj1	4.86	3.61	3.52	6.83	10.38	3.79
Csrp2	4.3	7.4	5.81	10.78	13.45	6.08
Ecm1	3.02	2.94	2.89	5.07	5.91	4.2
Eps8l2	1.86	2.28	2.73	4.53	5	2.28
Rsph1	7.11	7.44	6.53	11.88	17.39	6.83
Casp8	0.9	1.14	1.18	1.74	2.1	1.7
Col1a2	0.94	1.96	1.95	3.11	3.78	1.42
Cgn	1.31	1.17	1.89	2.25	2.89	2.43
Hba-a1	214.98	169.55	258.61	393.91	453.68	243.02
Sod3	6.15	5.88	6.53	10.61	14.11	6.92
Rab11fip1	0.45	0.49	0.64	1.09	1.09	0.58
Traip	12.3	11.43	13.53	20.49	19.65	21.39
Piezo1	1.42	1.57	2.05	3.37	3.14	1.93
Lama5	1.1	1.62	1.77	3.17	2.67	1.64
Car8	0.71	0.59	0.66	0.63	1.71	0.95
Mill2	0.71	1.25	0.99	1.71	1.73	2.09
Col6a1	22.53	18.84	25.8	24.81	51.63	35.94
Col1a1	1.09	1.35	1.48	1.93	2.81	1.8
Spint2	15.25	20.71	22.56	36.23	42.68	18.57
Ccdc114	1.48	1.37	1.51	2.72	3.25	1.42
Lrrk1	0.49	0.45	0.49	0.63	1.1	0.72
Bmp6	2.99	4.06	4.91	8.49	8.25	3.05
Hspb1	9.5	15.86	10.79	18.83	25.58	15.07
Crb2	1.19	0.99	1.22	2.14	2.3	1.16
Bmp7	2.04	3.38	3.61	4.9	6.67	3.31
Vstm4	1.27	1.25	1.29	2.24	2.3	1.68
Thbd	1.49	2.81	2.42	3.1	5.63	2.29
Tspo	8.54	8.64	7.26	13.8	16.84	8.97
Tent5a	1.01	0.43	0.99	1.43	1.44	1.08
Aldh1a2	1.99	3.12	3.44	4.49	6.6	2.76
Vamp8	13.92	24.47	18.3	33.41	42.4	15.54
Lepr	0.65	0.75	0.93	1.71	1.56	0.74
Cpq	4.24	5.8	5.61	9.71	10.05	5.39
Dab2	1.87	2.22	3.44	5.04	4.98	2.38
Steap2	1.18	1.18	1.38	2.6	2.38	1.09
Six5	1.88	2.28	2.02	3.74	3.73	2.4
Cldn5	19.09	27.59	21.6	31.85	45.55	31.47
Hes1	11.42	9.45	8.99	14.2	17.95	15.36
Sap25	16.21	16.61	15.6	24.8	30.08	21.6
Myof	0.84	0.89	1.16	1.62	1.99	0.97
Pla2g4b	2.62	2.34	2.29	3.38	3.88	4.15
Cp	1.76	2.47	2.71	3.06	4.81	2.82
Sowahc	2.41	2.47	3.59	5.64	5.44	2.28
Exosc6	6.4	8.33	6.03	8.85	12.46	11.28
Tbc1d2	1.24	1.39	1.71	3.09	2.29	1.88
Myh7	1	1.51	1.39	1.38	2.89	1.82
Lgals4	1.91	2.67	2.32	3.67	4.54	2.96
Anxa2	6.47	7.45	5.62	8.44	15.09	6.87
Spef1	3.14	4.15	4.41	4.91	8.07	5.33
Dok3	7.95	9.08	7	11.15	17.38	12.95
Igfbp3	6.7	7.84	8.58	9.03	16.16	10.72
Wtip	3.62	4.36	2.73	5.71	6.53	4.32
Serpinh1	6.92	9.28	8.44	12.75	16.36	9.13
Ifitm2	10.75	12.71	16.65	19.42	28.41	13.6
Col5a2	3.14	3.06	4.03	4.16	6.85	4.8
Cab39l	8.9	9.38	11.04	19.17	18.06	8.05
Cpne7	125	142.97	153.47	160.8	290.95	208.05

Fat4	2.42	2.9	2.31	1.98	1.15	2.05
Gm36722	0.98	0.74	0.71	0.39	0.51	0.82
Filip1	2.53	3.19	2.4	2.47	1.36	2.08
LOC118568634	10.74	6.77	9.84	6.51	3.87	7.89
Atp8a2	2.36	1.73	2.91	1.28	1.76	1.6
Bambi	3.91	3.7	2.68	2.7	1.91	2.23
Wnt9a	2.69	2.78	2.63	2.03	1.6	1.67
Pcdhb22	1.84	1.84	2.24	1.55	0.9	1.37
Sv2c	6.8	6.66	4.65	4.87	2.59	4.2
Hivep3	2.85	1.53	3.62	1.67	1.14	2.23
Xylt1	2.29	1.75	3.73	1.92	1.23	1.77
Npnt	8.66	9.34	5.36	6.39	2.94	5.39
Lnpep	2.08	0.92	2.6	1.19	1.13	1.17
Megf9	13.77	7.94	15.82	8.99	5.97	8.24
Fam205a4	0.94	0.68	1.17	0.55	0.72	0.55
Rskr	2.14	2.23	1.37	1.34	0.76	1.38
Parm1	11.09	6.96	16.42	7.8	5.01	7.95
Igfn1	2.79	2.09	1.21	0.95	1.97	1.8
Mmp16	5.02	4.93	4	3.29	2.35	2.68
Htr2a	1.35	0.94	2.11	1.11	0.54	0.9
Ankdd1a	0.94	1.37	0.63	0.74	0.3	0.56
Ttn	0.04	0.06	0.04	0.03	0.03	0.03
Fbxo48	0.82	0.84	0.57	0.44	0.27	0.31
LOC118568284	2.34	1.27	2.95	1.6	0.55	1.35
Tdo2	2.6	4.52	1.36	1.73	0.68	1.74
Gm46058	0.56	0.27	0.62	0.34	0.22	0.17
LOC118568301	0.79	1	0.97	0.35	0.16	0.74
Cdkl5	2.83	1.3	3.26	1.33	0.78	1.07
Kcnj2	3.64	3.95	3.38	1.83	1.16	1.67
Rspo1	2.91	1.59	0.75	0.64	0.88	0.58
Onecut1	0.82	0.57	0.28	0.33	0	0.08
Gm765	2.24	1.25	0.18	0.16	0.24	0.05
Htd2	0.24	0.29	0.27	0	0	0

Table S3. Heatmap of miRNA sequencing data in female blood

Gene ID	FB1	FB2	FB3	FB4	FB5	FB6
mmu-miR-8112	1.333	1.85	0.825	5.62	1.251	3.379
novel-mmu-miR29-5p	7.444	8.53	5.657	3.817	3.128	5.175
mmu-miR-206-3p	5.333	5.755	6.718	2.439	4.129	2.957

Table S4. Heatmap of miRNA sequencing data in female hippocampus

Gene ID	FH1	FH2	FH3	FH4	FH5	FH6
novel-mmu-miR249-5p	0.779	0.783	0.514	2.41	3.048	2.167
novel-mmu-miR143-3p	1.364	2.783	3.924	8.166	7.006	12.208
novel-mmu-miR142-3p	0.292	1.522	1.261	2.276	3.412	5.087
mmu-miR-466i-5p	0	0.217	0.747	1.651	1.183	1.548
mmu-miR-211-5p	6.526	7.132	13.173	15.262	57.688	12.606
novel-mmu-miR279-3p	0.146	0.174	0.42	0.803	0.955	0.973
novel-mmu-miR2-5p	0.584	0.478	0.561	0.714	2.866	1.504
novel-mmu-miR72-3p	0.195	0.087	0.14	0.357	0.773	0.619
novel-mmu-miR328-5p	1.997	4.175	2.336	7.676	5.505	9.598
novel-mmu-miR175-5p	0.195	0.043	0.327	1.026	1.365	0.133
novel-mmu-miR112-3p	1.023	1.522	2.382	1.874	4.641	5.396
mmu-miR-200b-5p	0.292	0.261	0.047	0.089	0.045	2.698
novel-mmu-miR15-5p	0.243	0.826	1.495	1.383	2.229	3.14
mmu-miR-6538	5.211	6.567	9.576	10.889	20.791	14.243
mmu-miR-182-5p	14.999	9.393	7.007	5.489	22.429	176.616

mmu-miR-3470a	0.73	0.522	0.234	0.848	1.274	1.194
mmu-miR-96-5p	6.087	8.828	2.943	1.964	22.702	135.702
novel-mmu-miR433-5p	0.195	0.696	0.701	1.026	0.864	1.813
mmu-miR-183-5p	24.496	8.35	12.332	5.043	33.848	439.882
novel-mmu-miR164-3p	0.39	0.565	0.467	0.714	1.137	1.194
mmu-miR-3470b	0.633	0.652	0.187	1.071	1.137	0.885
novel-mmu-miR214-3p	0.828	1.087	1.448	2.187	1.956	2.477
mmu-miR-429-3p	62.336	61.971	66.098	41.68	53.047	996.447
novel-mmu-miR186-5p	0.73	1.044	0.654	0.803	2.502	1.592
mmu-miR-200a-3p	16.46	19.091	16.256	8.836	16.97	325.102
novel-mmu-miR389-3p	0.682	1.261	2.663	3.302	2.457	3.671
mmu-miR-200b-3p	6.574	4.61	6.54	3.749	5.823	54.449
mmu-miR-200c-3p	4.091	1.479	1.308	0.491	5.095	48.655
novel-mmu-miR421-5p	1.656	0.043	1.495	2.231	2.093	2.433
novel-mmu-miR202-3p	1.412	1.261	1.775	2.856	3.367	1.946
mmu-miR-223-3p	0.682	1.609	1.542	2.678	2.366	1.238
novel-mmu-miR24-5p	2.24	3.349	1.915	5.266	3.185	2.61
mmu-miR-148a-3p	10.178	8.741	5.372	6.248	7.279	18.798
novel-mmu-miR380-5p	8.084	6.132	2.522	8.568	8.462	5.662
mmu-miR-320-3p	53.034	45.837	42.602	57.254	56.005	64.224
mmu-miR-145a-5p	30.729	46.532	32.979	46.544	58.643	29.547
mmu-miR-30a-3p	14.074	11.307	9.996	16.199	12.42	9.864
novel-mmu-miR120-3p	19.967	11.046	11.585	7.854	6.597	6.015
mmu-let-7i-3p	99.98	81.41	56.896	54.532	35.668	26.495
mmu-miR-378c	5.649	5.175	4.157	2.99	2.366	1.858
mmu-miR-135b-5p	301.451	328.292	152.936	173.503	112.146	83.421
mmu-miR-196a-5p	0.097	0.217	0.234	0	0	0
mmu-miR-3965	3.019	1.783	2.289	0.491	0.182	0.265
novel-mmu-miR274-5p	5.26	3.914	5.512	0.268	0.318	0.088
